# Association between knee osteoarthritis and the risk of cardiovascular disease and the synergistic adverse effects of lack of exercise

**DOI:** 10.1038/s41598-023-29581-1

**Published:** 2023-02-16

**Authors:** Dojoon Park, Yong-Moon Park, Seung-Hyun Ko, Youn-Ho Choi, Dong-Uk Min, Jae-Hyun Ahn, Bongseong Kim, Hae-Seok Koh, Kyungdo Han

**Affiliations:** 1 Department of Orthopedic Surgery, St. Vincent’s Hospital, College of Medicine, The Catholic University of Korea, 93, Jungbu-daero, Paldal-gu, Suwon, Gyeonggi-do Republic of Korea; 2 Department of Epidemiology, Fay W. Boozman College of Public Health, University of Arkansas for Medical Sciences, Little Rock, AR USA; 3 Department of Internal Medicine, St. Vincent’s Hospital, College of Medicine, The Catholic University of Korea, Seoul, Republic of Korea; 4 Department of Statistics and Actuarial Science, Soongsil University, Seoul, Republic of Korea

**Keywords:** Diseases, Health care, Medical research, Rheumatology

## Abstract

We aimed to determine whether knee OA is associated with CVD risk and all-cause death and to evaluate whether the association differs by exercise behavior. We used Korea National Health Insurance Service (KNHIS) database and included 201,466 participants (7572 subjects diagnosed with knee OA) who underwent health screening between 2009 and 2015. Those who had been diagnosed with knee OA or CVD before the index year were excluded. Cox proportional hazard models were used after adjusting for sociodemographic and CVD risk factors to evaluate the association between knee OA and CVD risk and all-cause death. Stratification analysis was further performed to determine the effect of exercise behavior on this relationship. During a median follow-up of 7.06 ± 2.24 years, 8743 CVD (2510 MI and 6553 stroke) cases developed. Individuals with knee OA had increased risks of CVD [hazard ratio (HR) 1.26, 95% confidence interval (CI) 1.15–1.38], myocardial infarction (MI) (HR 1.20, 95% CI 1.00–1.44), and stroke (HR 1.29, 95% CI 1.16–1.43) compared with those without knee OA. Those with knee OA who did not exercise had an increased risk of CVD (HR 1.25, 95% CI 1.11–1.40), whereas no significant increased CVD risk was observed in those with knee OA who exercised at least once a week (HR 1.11, 95% CI 0.96–1.28). There was no association between knee osteoarthritis and all-cause death. Knee OA was independently associated with an increased risk of CVD. Lack of exercise might have a synergistic adverse effect on the association between knee OA and CVD.

## Introduction

Osteoarthritis (OA) is the second most prevalent chronic disease worldwide after hypertension^1,2^. Radiological findings of OA are represented in 80% of the population aged 65 or older, and more than 60% complain of symptoms^3^. Particularly, knee joint is the most common site where OA occurs due to large weight loads^4,5^. Knee OA is characterized by pain, impaired function, and impoverished quality of life, and is one of the main causes of disability in the elderly^6^. Knee OA is also a leading cause of medical expenses and morbidity^7^.

Cardiovascular diseases (CVD), such as myocardial infarction (MI) and stroke, are the main causes of mortality in the world's population and are supposed to increase to 23.3 million by 2030^8^. CVD is a great burden personally, socially, and economically^9^. Therefore, identification of risk factors of CVD and searching for interventions for prevention are very imperative.

The conventional risk factors for CVD, such as age, hypertension, diabetes, obesity, and low physical activity^5,10–13^ are also associated with the development and progression of knee OA^14,15^. Decreased physical activity^16^ and muscle weakness^17^, which are common features of patients with knee OA, are also known to be associated with an increased risk of CVD and mortality^18–20^. Shared pathophysiological pathways and risk factors suggest an association between the two prevalent diseases.

Given the pervasiveness and social burden of knee OA and CVD in the general population, understanding the relationship between both diseases is important for clinical and public health. However, as stated in a previous systematic review, there are few large cohort studies evaluating the CVD risk in patients with knee OA^21^, particularly in Asians. In addition, whether exercise behavior effects on the association between knee OA and CVD outcome, and the extent of its effect is more unclear. This study was conducted to determine the risk of CVD including MI, stroke, and all-cause death in patients with knee OA using the National Health Insurance data and to evaluate the effect of exercise behavior on these associations.

## Methods

The National Health Insurance Service (NHIS) is a single insurance company that covers about 97% of the total population of Korea^22^. We collected data from January 1, 2010 to December 31, 2019 from the National Health Insurance claim database, and this database is linked with the health examination database established by the National Health Insurance Service.

The NHIS database contains medical claim data such as diagnosis, and prescription, and the diagnosis is coded according to the International Classification of Diseases, 10th revision (ICD-10). It is also recommended that insured subjects undergo a general health check-up every 2 years, which includes demographic information and self-report questionnaires on health-related behaviors such as exercise habits, smoking status, and alcohol consumption. The validity of the NHIS cohort is confirmed in previous studies^23–25^.

### Study population

**Figure 1:**
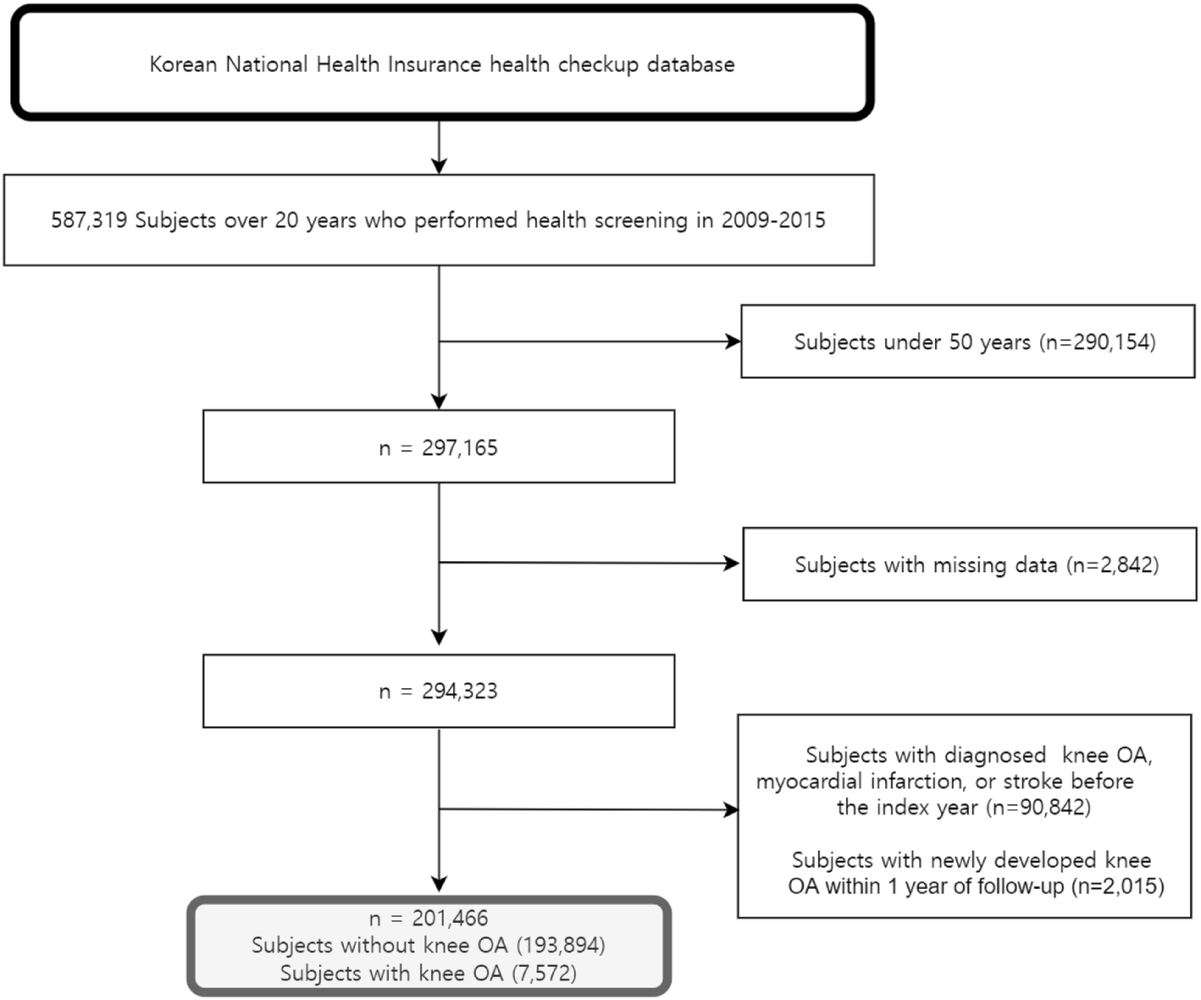
Flow chart of the cohort selection. *OA* osteoarthritis.

This study analyzed 297,165 people over the age of 50 years who underwent a general health checkup from 2009 to 2015. Since most osteoarthritis occurs after the age of 50 years, this age was determined as the cut-off^49^. Subjects diagnosed with knee OA, MI, or stroke before the index year (enrollment year) and those with knee OA within 1 year from the index year were excluded to minimize reversed causality. After excluding those with missing values, 201,466 subjects were included in the final analysis (Fig. 1). The mean follow-up period for MI or stroke and all-cause death was 7.06 ± 2.24 years and 7.22 ± 2.12 years, respectively.

### Ascertainment of knee OA and outcomes

Based on a previous validation study^26^, knee OA was defined by knee OA ICD-10 code (M17) or any site OA code (M15, polyarthrosis or M19, other arthrosis) in combination with a procedure for a knee X-ray in the same claim.

The primary endpoint was the diagnosis of MI (ICD‐10 codes I21‐24) or stroke (ICD‐10 codes G45‐46). MI was defined as one or more ICD-10 codes for acute MI (I21-22), accompanied by hospitalization or more than two outpatient clinic visits. Stroke was defined as the corresponding ICD-10 code (I63-43) for diagnoses during hospitalization as assessed by a brain imaging study (e.g., computed tomography, magnetic resonance imaging). The validity of these definitions has been confirmed in previous studies^24,25^. Diagnosed MI or stroke was defined as the onset of CVD. The secondary endpoint was all-cause death in both the knee OA and the comparison cohorts. Incidence of all-cause death was analyzed in both groups.

### Measurement of general health behaviors and comorbidities

Information on lifestyle-related factors was collected using the questionnaires. Income status was classified into quintiles based on the annual insurance premium to the KNHIS. The categories of smoking status were as follows: never, former and current smoker. Drinking status was categorized as: none, moderate (1–< 30 g/day), and heavy (≥ 30 g/day). Exercise behavior was stratified into exercise and regular exercise according to frequency, intensity, and duration. The definition of exercise is (1) moderate physical activity for > 30 min, ≥ 1 time/week or (2) vigorous physical activity for > 20 min, ≥ 1 time/week. And regular exercise is (1) moderate physical activity for > 30 min, ≥ 5 times/week or (2) vigorous physical activity for > 20 min, ≥ 3 times/week^27^.

Comorbidities were defined by a previously verified methodology^28,29^. Hypertension, type 2 diabetes mellitus (T2DM), and dyslipidemia were defined using a combination of the ICD-10 code and claims data of associated drugs or the measurements of the health check-up. Details of the definition of comorbidities considered in this study are presented in Table S1. After fasting for at least 8 h from midnight, blood collection was done to confirm the concentration of glucose and creatine, and lipid profile.

### Statistical analysis

Continuous variables were presented as mean ± standard deviation, and categorical variables were presented as numbers and percentages. For comparison between cohorts, a Student test was used for continuous variables and a chi-square test was used for categorical variables. The incidence of CVD, MI, stroke, and all-cause death were presented per 1000 person-years. The cumulative probability of occurrence of CVD, MI, stroke, and all-cause death were plotted as a Kaplan–Meier curve and compared using the log-rank test.

A multivariable Cox regression model was used to calculate hazard ratios (HRs) and 95% confidence intervals (CIs) for the associations of knee OA with CVD and mortality. Proportional hazard assumptions were evaluated by Schoenfeld residuals with the logarithm of the cumulative hazards function. No significant departures from proportionality in hazards over time were detected. Potential confounders or effect modifiers were identified a priori based on the literature review. The following covariates at baseline were included in multivariable-adjusted models: age, sex, income status, hypertension, dyslipidemia, smoking, drinking alcohol, exercise, body mass index (BMI), glucose, and glomerular filtration rate (GFR). Potential effect modification was evaluated with likelihood ratio tests for age (< 65 years, over 65 years), sex, T2DM, hypertension, dyslipidemia, chronic kidney disease (CKD), and BMI (< 25 kg/m^2^, over 25 kg/m^2^). Stratification analysis was additionally performed according to the combination of sex and age group (men under 65 years, men over 65 years, women under 65 years, and women over 65 years) and exercise behavior. According to the reviewer's suggestions, an additional sensitivity analysis was performed for participants with MI or stroke only.

The p values provided are two-sided, with the level of significance at 0.05. All statistical analysis procedures were performed with SAS version 9.4 (SAS Institute, Cary, NC, USA).

### Ethical approval

The entire process of this study complied with the ethical norms of the Declaration of Helsinki. This study was approved by both the KNHIS and the IRB of the Catholic University of Korea (IRB No. VC22ZISI0163), and informed consent was exempted by the IRB of the Catholic University of Korea due to the retrospective nature of the study and the anonymity of the data.

## Results

### Baseline characteristics

The baseline characteristics of participants with and without knee OA are summarized in Table 1. Compared to individuals without knee OA, those with knee OA cohort were older and more females. Those with knee OA were more likely to be accompanied by hypertension, T2DM, and dyslipidemia than comparison cohort (44.3% vs 36.5%, 14.2% vs 13.2% and 32.3% vs 25.8%; p < 0.0001, p = 0.0143, p < 0.0001, respectively). The knee OA cohort was more likely to be fewer smokers, less alcohol consumption, and more the lowest quintile house income. There was no statistically significant discrepancy between the two groups regarding the percentage of regular exercise.

**Table 1:** Baseline characteristics of study participants according to knee OA.

	Knee osteoarthritis		p value
	No (n = 193,894)	Yes (n = 7572)	
Male, n (%)	104,257 (53.77)	2467 (32.58)	< 0.0001
Age, mean	56.89 ± 7.6	59.99 ± 8.46	< 0.0001
Low income < 20%, n (%)	32,762 (16.9)	1414 (18.67)	< 0.0001
Type 2 DM, n (%)	25,591 (13.2)	1073 (14.17)	0.0143
Hypertension, n (%)	70,833 (36.53)	3353 (44.28)	< 0.0001
Dyslipidemia, n (%)	49,922 (25.75)	2446 (32.3)	< 0.0001
Smoking, n (%)			< 0.0001
Non	117,525 (60.61)	5781 (76.35)	
Ex	34,589 (17.84)	883 (11.66)	
Current	41,780 (21.55)	908 (11.99)	
Alcohol, n (%)			< 0.0001
Non	111,951 (57.74)	5360 (70.79)	
Mild	66,173 (34.13)	1828 (24.14)	
Heavy	15,770 (8.13)	384 (5.07)	
Exercise, n (%)	98,334 (50.71)	3320 (43.84)	< 0.0001
Regular exercise, n (%)	41,056 (21.17)	1536 (20.29)	0.063
CKD, n (%)	11,764 (6.07)	596 (7.87)	< 0.0001
BMI, kg/m^2^	23.86 ± 2.97	24.4 ± 3.16	< 0.0001
Glucose, mg/dL	101.97 ± 27.03	101.07 ± 25.84	0.0046
SBP, mmHg	124.87 ± 15.55	125.86 ± 15.8	< 0.0001
DBP, mmHg	77.6 ± 10.23	77.42 ± 10.06	0.1407
Total cholesterol, mg/dL	201.36 ± 37.75	204.17 ± 38.44	< 0.0001
HDL-C, mg/dL	55.06 ± 21.69	55.62 ± 22.07	0.0271
LDL-C, mg/dL	119.53 ± 36.95	121.77 ± 37.02	< 0.0001
e-GFR, mL/min/1.73 m^2^	86.75 ± 37.93	86.35 ± 36.56	0.3667

Continuous variables were presented using mean and standard deviation. Categorical variables are expressed in numbers and percentages.

*DM* diabetes mellitus, *CKD* chronic kidney disease, *BMI* body mass index, *SBP* systolic blood pressure, *DBP* diastolic blood pressure, *HDL* high-density lipoprotein cholesterol, *LDL-C* low-density lipoprotein cholesterol, *e-GFR* estimated glomerular filtration rate.

### Association of knee OA with incidence and risk of CVD and all-cause death

**Figure 2:**
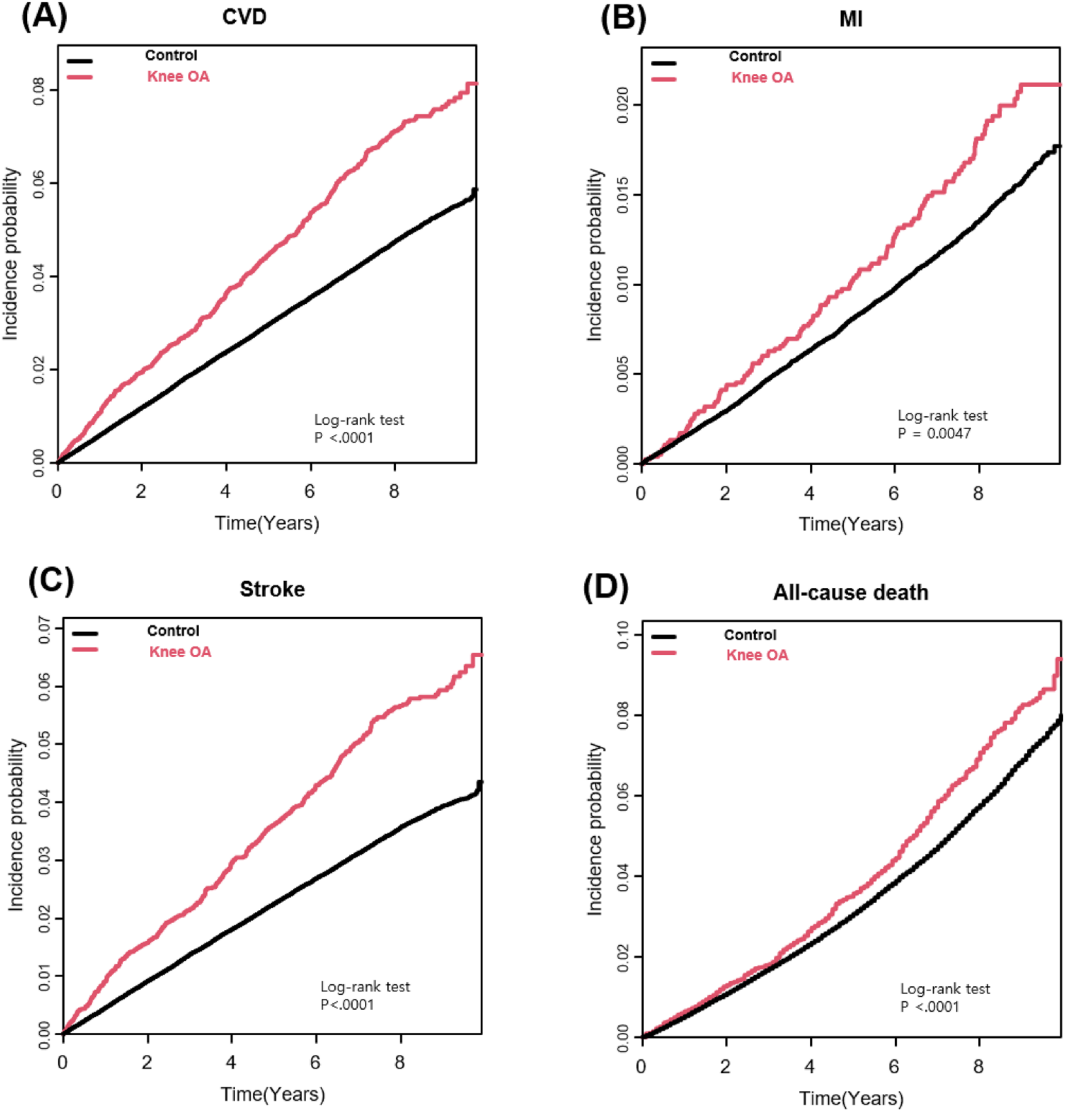
Comparing (**A**) cardiovascular events, (**B**) myocardial infarction, (**C**) stroke and (**D**) all-cause death between knee osteoarthritis and control cohorts with a log-rank p value. *CVD* cardiovascular disease, *MI* myocardial infarction, *OA* osteoarthritis.

The Knee OA group had a higher incidence of CVD, MI, stroke, and all-cause death than the comparison group (incidence rate, 1000 person-years; CVD: 8.96 vs 6.04, MI: 2.23 vs 1.71, stroke: 7.05 vs 4.48, all-cause death: 8.98 vs 7.38, respectively). The Kaplan Meyer curve determined that the knee OA cohort had a significantly higher cumulative incidence of CVD, MI, stroke, and all-cause deaths than the comparison group (Fig. 2; log-rank p < 0.0001, p = 0.0047, p < 0.0001, and p < 0.0001, respectively). In the crude analyses, individuals with knee OA had a significantly higher risk of all CVD, MI, stroke, and all-cause death compared with without knee OA cohort. After multivariable adjustments for 11 confounding factors, the positive associations remained significant for incidents all CVD (HR 1.26, 95% CI 1.15–1.38), MI (HR 1.20, 95% CI 1.00–1.44), and stroke (HR 1.29, 95% CI 1.16–1.43). However, the risk of all-cause death was not statistically different between the two groups (HR 1.01, 95% CI 0.92–1.10). Details are given in Table 2.

**Table 2:** Risk of cardiovascular events, myocardial infarction, stroke, and all-cause death in patients with knee OA and control cohorts.

	N	Event	Duration, person-years	IR (per 1000)	HR (95% CI)		
					Model 1	Model 2	Model 3
CVD (MI or stroke)							
Controls	193,894	8247	1,366,461	6.04	1 (ref.)	1 (ref.)	1 (ref.)
Knee OA	7572	496	55,347	8.96	1.49 (1.36, 1.63)	1.28 (1.16, 1.40)	1.26 (1.15, 1.38)
MI							
Controls	193,894	2383	1,389,789	1.71	1 (ref.)	1 (ref.)	1 (ref.)
Knee OA	7572	127	56,920	2.23	1.29 (1.08, 1.55)	1.22 (1.02, 1.46)	1.20 (1.00, 1.44)
Stroke							
Controls	193,894	6160	1,373,673	4.48	1 (ref.)	1 (ref.)	1 (ref.)
Knee OA	7572	393	55,732	7.05	1.58 (1.42, 1.75)	1.30 (1.17, 1.44)	1.29 (1.16, 1.43)
All**-**cause death							
Controls	193,894	10,320	1,397,766	7.38	1 (ref.)	1 (ref.)	1 (ref.)
Knee OA	7572	515	57,356	8.98	1.20 (1.09, 1.31)	0.98 (0.89, 1.07)	1.01 (0.92, 1.10)

Model 1: no adjustment; Model 2: adjusted for age and sex; Model 3 : adjusted for age, sex, income, hypertension, dyslipidemia, smoking, drinking, exercise, BMI, glucose, and GFR.

*OA* osteoarthritis, *CVD* cardiovascular disease, *MI* myocardial infarction, *IR* incidence rate, *HR* hazard ratio, *CI* confidence interval.

In the sensitivity analysis conducted on participants with MI or stroke only, stroke was still found to have a significant association with knee OA (HR 1.27, 95% CI 1.14–1.41). However for MI, the difference between the knee OA and comparison cohorts, which was confirmed in the crude analysis (HR 1.22, 95% CI 1.01–1.49), was not confirmed after adjusting for 11 confounding variables (HR 1.16, 95% CI 0.96–1.42) (S2 Table).

Stratified analyses by potential modifiers of the association of knee OA with risk of CVD and all-cause mortality are shown in Table 3. Age was a borderline significant moderator of the effects of knee OA on CVD (p for interaction = 0.071), with the adverse interaction effect in younger over older (age < 65; HR 1.38, 95% CI 1.21–1.58; age ≥ 65: HR 1.17, 95% CI 1.03–1.32, respectively). Sex did not statistically moderate the effects of knee OA on CVD (p for interaction = 0.408). There was no evidence of effect modification by age and sex for MI, stroke, and all-cause death. As for comorbid diseases, a significant modifying effect of T2DM was observed on the MI risk by knee OA (p for interaction = 0.021). Although not statistically significant, the HR estimate of MI was lower in the knee OA cohort without T2DM than the knee OA cohort with T2DM. There was no statistically significant trend (p for interaction = 0.412), the risk of CVD and MI was higher in the knee OA cohort with hypertension (HR 1.33, 95% CI 1.18–1.49; HR 1.27, 95% CI 1.01–1.60, respectively) compared to the knee OA cohort without hypertension. All HR values were adjusted for age, sex, income, hypertension, dyslipidemia, smoking, drinking, exercise, BMI, glucose, and GFR.

**Table 3:** Subgroup analysis of association between knee OA and the risk of cardiovascular events, myocardial infarction, stroke, and all-cause death according to age, sex, type 2 diabetes mellitus, hypertension, dyslipidemia, chronic kidney disease, and body mass index.

Subgroup	IR^a^	CVD (MI or stroke)		MI			Stroke			All-cause death		
		HR (95% CI)^b^	P_int_	IR^a^	HR (95% CI)^b^	P_int_	IR^a^	HR (95% CI)^b^	P_int_	IR^a^	HR (95% CI)^b^	P_int_
Age < 65			0.071			0.289			0.193			0.327
Controls	4.17	1 (ref.)		1.29	1 (ref.)		2.95	1 (ref.)		3.73	1 (ref.)	
Knee OA	5.69	1.38 (1.21, 1.58)		1.60	1.33 (1.03, 1.71)		4.14	1.39 (1.19, 1.63)		3.68	1.09 (0.92, 1.28)	
Age ≥ 65												
Controls	15.59	1 (ref.)		3.81	1 (ref.)		12.30	1 (ref.)		25.37	1 (ref.)	
Knee OA	17.02	1.17 (1.03, 1.32)		3.73	1.09 (0.85, 1.41)		14.18	1.21 (1.06, 1.39)		21.45	0.99 (0.89, 1.09)	
Male			0.408			0.440			0.680			0.426
Controls	7.46	1 (ref.)		2.30	1 (ref.)		5.36	1 (ref.)		10.13	1 (ref.)	
Knee OA	12.73	1.32 (1.15, 1.51)		3.73	1.28 (1.01, 1.64)		9.35	1.32 (1.13, 1.55)		15.51	1.04 (0.93, 1.18)	
Female												
Controls	4.42	1 (ref.)		1.04	1 (ref.)		3.49	1 (ref.)		4.24	1 (ref.)	
Knee OA	7.24	1.22 (1.08, 1.38)		1.54	1.11 (0.86, 1.45)		6.00	1.27 (1.10, 1.45)		5.95	0.97 (0.85, 1.11)	
Type 2 DM (−)			0.982			0.021			0.278			0.360
Controls	5.34	1 (ref.)		1.51	1 (ref.)		3.96	1 (ref.)		6.45	1 (ref.)	
Knee OA	7.88	1.26 (1.14, 1.40)		2.18	1.34 (1.10, 1.64)		5.98	1.25 (1.11, 1.41)		7.61	0.99 (0.89, 1.09)	
Type 2 DM (+)												
Controls	10.74	1 (ref.)		3.12	1 (ref.)		8.07	1 (ref.)		13.64	1 (ref.)	
Knee OA	15.84	1.26 (1.04, 1.52)		2.52	0.76 (0.48, 1.18)		13.90	1.42 (1.16, 1.73)		17.43	1.08 (0.91, 1.28)	
Hypertension (−)			0.165			0.412			0.256			0.313
Controls	4.39	1 (ref.)		1.33	1 (ref.)		3.16	1 (ref.)		5.45	1 (ref.)	
Knee OA	5.65	1.16 (0.99, 1.35)		1.48	1.09 (0.81, 1.46)		4.31	1.19 (1.00, 1.42)		6.69	1.07 (0.93, 1.22)	
Hypertension (+)												
Controls	8.86	1 (ref.)		2.37	1 (ref.)		6.75	1 (ref.)		10.66	1 (ref.)	
Knee OA	13.23	1.33 (1.18, 1.49)		3.17	1.27 (1.01, 1.60)		10.56	1.35 (1.19, 1.53)		11.83	0.97 (0.87, 1.09)	
Dyslipidemia (−)			0.785			0.519			0.747			0.197
Controls	5.82	1 (ref.)		1.59	1 (ref.)		4.38	1 (ref.)		7.69	1 (ref.)	
Knee OA	8.73	1.27 (1.14, 1.42)		2.15	1.25 (1.00, 1.56)		6.84	1.27 (1.12, 1.45)		10.01	1.05 (0.94, 1.16)	
Dyslipidemia (+)												
Controls	6.67	1 (ref.)		2.06	1 (ref.)		4.79	1 (ref.)		6.49	1 (ref.)	
Knee OA	9.46	1.24 (1.06, 1.45)		2.40	1.11 (0.82, 1.50)		7.49	1.32 (1.11, 1.58)		6.82	0.91 (0.76, 1.09)	
CKD (−)			0.838			0.539			0.878			0.618
Controls	5.65	1 (ref.)		1.61	1 (ref.)		4.19	1 (ref.)		6.76	1 (ref.)	
Knee OA	8.28	1.27 (1.15, 1.40)		2.10	1.23 (1.01, 1.49)		6.45	1.29 (1.15, 1.44)		8.15	1.02 (0.93, 1.13)	
CKD (+)												
Controls	11.79	1 (ref.)		3.21	1 (ref.)		8.93	1 (ref.)		16.53	1 (ref.)	
Knee OA	16.96	1.23 (0.97, 1.56)		3.68	1.04 (0.64, 1.70)		14.04	1.31 (1.01, 1.70)		18.35	0.96 (0.77, 1.19)	
BMI < 25			0.430			0.272			0.959			0.311
Controls	5.95	1 (ref.)		1.65	1 (ref.)		4.47	1 (ref.)		8.18	1 (ref.)	
Knee OA	8.78	1.22 (1.09, 1.38)		2.00	1.10 (0.86, 1.40)		7.19	1.29 (1.13, 1.47)		10.24	0.98 (0.88, 1.09)	
BMI ≥ 25												
Controls	6.20	1 (ref.)		1.84	1 (ref.)		4.51	1 (ref.)		5.81	1 (ref.)	
Knee OA	9.24	1.32 (1.14, 1.52)		2.57	1.34 (1.03, 1.75)		6.84	1.29 (1.10, 1.53)		7.12	1.08 (0.93, 1.27)	

*OA* osteoarthritis, *CVD* cardiovascular disease, *MI* myocardial infarction, *IR* incidence rate, *HR* hazard ratio, *CI* confidence interval, *DM* diabetes mellitus, *CKD* chronic kidney disease, *BMI* body mass index.

^a^Per 1000 person‐years.

^b^The HR was adjusted for adjusted for age, sex, income, hypertension, dyslipidemia, smoking, drinking, exercise, BMI, glucose, and GFR.

In analyzes stratified by sex and age combination, the HR of CVD of knee OA cohort was higher in males under 65 than in females under 65 (Table 4; HR 1.50, 95% CI 1.23–1.83; HR 1.32, 95% CI 1.10–1.59, respectively), although the trends were not statistically significant.

**Table 4:** The risk of cardiovascular events, myocardial infarction, stroke, and all-cause death according to the knee osteoarthritis and combination of age and sex.

Subgroup	CVD (MI or Stroke)			MI			Stroke			All-cause death		
	IR^a^	HR (95% CI)^b^	P_int_	IR^a^	HR (95% CI)^b^	P_int_	IR^a^	HR (95% CI)^b^	P_int_	IR^a^	HR (95% CI)^b^	P_int_
Age < 65, male			0.149			0.305			0.658			0.246
Controls	5.30	1 (ref.)		1.85	1 (ref.)		3.55	1 (ref.)		5.48	1 (ref.)	
Knee OA	8.69	1.50 (1.23, 1.83)		3.03	1.50 (1.08, 2.09)		5.54	1.43 (1.11, 1.82)		7.09	1.18 (0.95, 1.46)	
Age ≥ 65, male												
Controls	17.55	1 (ref.)		4.37	1 (ref.)		13.77	1 (ref.)		31.09	1 (ref.)	
Knee OA	20.93	1.20 (0.99, 1.44)		5.08	1.15 (0.80, 1.65)		17.04	1.25 (1.02, 1.52)		31.79	1.02 (0.88, 1.17)	
Age < 65, female												
Controls	2.92	1 (ref.)		0.68	1 (ref.)		2.28	1 (ref.)		1.80	1 (ref.)	
Knee OA	4.43	1.32 (1.10, 1.59)		0.99	1.26 (0.86, 1.85)		3.56	1.362 (1.11, 1.67)		2.23	1.08 (0.84, 1.40)	
Age ≥ 65, female												
Controls	12.98	1 (ref.)		3.06	1 (ref.)		10.33	1 (ref.)		17.66	1 (ref.)	
Knee OA	14.83	1.12 (0.95, 1.33)		2.97	0.94 (0.65, 1.35)		12.57	1.20 (1.00, 1.44)		15.58	0.90 (0.77, 1.06)	

*OA* osteoarthritis, *CVD* cardiovascular disease, *MI* myocardial infarction, *IR* incidence rate, *HR* hazard ratio, *CI* confidence interval.

^a^Per 1000 person‐years.

^b^The HR was adjusted for adjusted for age, sex, income, hypertension, dyslipidemia, smoking, drinking, exercise, BMI, glucose, and GFR.

Stratified analyses by the exercise behavior are shown in Table 5. Individuals with knee OA who did not exercise had an increased risk of CVD (HR 1.25, 95% CI 1.11–1.40), compared to those without knee OA who did not exercise. A similar association was observed using the presence or absence of regular exercise (HR 1.27, 95% CI 1.15–1.41). In terms of stroke, a similar increase in risk was confirmed in the knee OA cohort who did not exercise compared to the control group who did not exercise (HR 1.28, 95% CI 1.13–1.46), and the same was found with or without regular exercise (HR 1.31, 95% CI 1.17–1.47). In contrast, there was no significant increase in CVD risk among those who exercised at least once a week or those reported to have regular exercise, compared to those without knee OA who did not exercise (HR 1.11, 95% CI 0.96–1.28; HR 1.13, 95% CI 0.92–1.40, respectively). In terms of the risk of all-cause death, there were no differences from the comparison group in whether knee OA patients had a behavior of exercise or regular exercise.

**Table 5:** The risk of cardiovascular events, myocardial infarction, stroke, and all-cause death according to the knee osteoarthritis and exercise behavior.

	Exercise behavior	N	Event	Duration, person-years	IR^a^	HR (95% CI)		
						Model 1	Model 2	Model 3
CVD (MI or stroke**)**								
Controls	Exercise (−)	95,560	4620	672,794	6.87	1 (ref.)	1 (ref.)	1 (ref.)
	Exercise (+)	98,334	3627	693,667	5.23	0.76 (0.73, 0.80)	0.84 (0.81, 0.88)	0.87 (0.84, 0.91)
Knee OA	Exercise (−)	4252	312	30,932	10.09	1.47 (1.31, 1.65)	1.27 (1.13, 1.42)	1.25 (1.11, 1.40)
	Exercise (+)	3320	184	24,416	7.54	1.10 (0.95, 1.27)	1.08 (0.93, 1.25)	1.11 (0.96, 1.28)
MI								
Controls	Exercise (−)	95,560	1331	685,735	1.94	1 (ref.)	1 (ref.)	1 (ref.)
	Exercise (+)	98,334	1052	704,054	1.49	0.77 (0.71, 0.84)	0.81 (0.74, 0.87)	0.85 (0.78, 0.92)
Knee OA	Exercise (−)	4252	80	31,941	2.50	1.29 (1.03, 1.61)	1.22 (0.97, 1.53)	1.20 (0.95, 1.50)
	Exercise (+)	3320	47	24,980	1.88	0.96 (0.72, 1.29)	0.98 (0.73, 1.31)	1.01 (0.76, 1.35)
Stroke								
Controls	Exercise (−)	95,560	3459	676,768	5.11	1 (ref.)	1 (ref.)	1 (ref.)
	Exercise (+)	98,334	2701	696,905	3.88	0.76 (0.72, 0.80)	0.86 (0.82, 0.91)	0.89 (0.84, 0.94)
Knee OA	Exercise (−)	4252	249	31,178	7.99	1.57 (1.38, 1.78)	1.30 (1.14, 1.48)	1.28 (1.13, 1.46)
	Exercise (+)	3320	144	24,554	5.86	1.15 (0.97, 1.36)	1.12 (0.94, 1.32)	1.15 (0.97, 1.35)
All-cause death								
Controls	Exercise (−)	95,560	6271	690,121	9.09	1 (ref.)	1 (ref.)	1 (ref.)
	Exercise (+)	98,334	4049	707,646	5.72	0.63 (0.61, 0.66)	0.75 (0.72, 0.79)	0.80 (0.77, 0.84)
Knee OA	Exercise (−)	4252	325	32,218	10.09	1.09 (0.98, 1.22)	0.91 (0.81, 1.02)	0.94 (0.84, 1.06)
	Exercise (+)	3320	190	25,138	7.56	0.82 (0.71, 0.95)	0.83 (0.72, 0.96)	0.91 (0.79, 1.05)
CVD (MI or stroke)								
Controls	Regular exercise (−)	152,838	6548	1,073,044	6.10	1 (ref.)	1 (ref.)	1 (ref.)
	Regular exercise (+)	41,056	1699	293,417	5.79	0.95 (0.90, 1.00)	0.91 (0.86, 0.96)	0.93 (0.88, 0.98)
Knee OA	Regular exercise (−)	6036	406	43,872	9.25	1.52 (1.37, 1.68)	1.29 (1.16, 1.42)	1.27 (1.15, 1.41)
	Regular exercise (+)	1536	90	11,475	7.84	1.29 (1.05, 1.58)	1.11 (0.90, 1.36)	1.13 (0.92, 1.40)
MI								
Controls	Regular exercise (−)	152,838	1889	1,091,411	1.73	1 (ref.)	1 (ref.)	1 (ref.)
	Regular exercise (+)	41,056	494	298,378	1.66	0.95 (0.86, 1.05)	0.89 (0.81, 0.98)	0.93 (0.84, 1.03)
Knee OA	Regular exercise (−)	6036	101	45,158	2.24	1.28 (1.05, 1.57)	1.21 (0.99, 1.47)	1.18 (0.97, 1.45)
	Regular exercise (+)	1536	26	11,762	2.21	1.266 (0.86, 1.86)	1.15 (0.78, 1.70)	1.18 (0.80, 1.73)
Stroke								
Controls	Regular exercise (−)	152,838	4888	1,078,739	4.53	1 (ref.)	1 (ref.)	1 (ref.)
	Regular exercise (+)	41,056	1272	294,934	4.31	0.95 (0.90, 1.01)	0.92 (0.87, 0.98)	0.94 (0.88, 0.99)
Knee OA	Regular exercise (−)	6036	326	44,178	7.38	1.63 (1.46, 1.83)	1.33 (1.19, 1.49)	1.31 (1.17, 1.47)
	Regular exercise (+)	1536	67	11,554	5.80	1.28 (1.01, 1.63)	1.085 (0.85, 1.38)	1.11 (0.87, 1.41)
All-cause death								
Controls	Regular exercise (−)	152,838	8411	1,097,684	7.66	1 (ref.)	1 (ref.)	1 (ref.)
	Regular exercise (+)	41,056	1909	300,082	6.36	0.83 (0.79, 0.87)	0.78 (0.74, 0.82)	0.82 (0.78, 0.86)
Knee OA	Regular exercise (−)	6036	425	45,507	9.34	1.20 (1.09, 1.32)	0.96 (0.87, 1.06)	0.99 (0.90, 1.10)
	Regular exercise (+)	1536	90	11,849	7.60	0.97 (0.79, 1.19)	0.81 (0.66, 0.10)	0.90 (0.73, 1.11)

Model 1: no adjustment; Model 2: adjusted for age and sex; Model 3 : adjusted for age, sex, income, hypertension, dyslipidemia, smoking, drinking, exercise, BMI, glucose, and GFR.

*OA* osteoarthritis, *CVD* cardiovascular disease, *MI* myocardial infarction, *IR* incidence rate, *HR* hazard ratio, *CI* confidence interval;

^a^Per 1000 person‐years.

## Discussion

To the best of our knowledge, this is the first longitudinal, large-scale, population-based cohort study to investigate the association between knee OA and CVD using national health insurance data in Asian populations. Our main results are as follows: (1) knee OA was associated with an increased risk of CVD after adjusting for a wide range of potential confounders. (2) In the subgroup analysis, there was a higher risk of cardiovascular complications in those with knee OA under 65 years of age. (3) individuals with knee OA who did not exercise had an increased risk of developing cardiovascular complications, but the CVD risk of those with knee OA who exercised at least once a week or regularly was not different from those without knee OA who did not exercise.

In Korea, knee OA ranks fifth in the number of outpatients over the age of 60 (third among chronic diseases), and the total treatment cost of outpatient and inpatient care reached almost 1.14 billion dollars in 2020^30^. In addition, deaths from CVD account for 19.2% of the total causes of death in Korea^31^. Therefore, it is very important to understand the relationship between knee OA and CVD, which is gaining importance in the elderly population.

The association between knee OA and CVD remains controversial, but many factors suggest that OA may be associated with increased CVD risk. First, OA and CVDs both share several risk factors. The association between OA and traditional cardiovascular risk factors such as hypertension^32^, diabetes^33^, dyslipidemia^34^, and obesity^35^ has been confirmed through several epidemiological studies. Second, NSAIDs, known to increase the risk of vascular events, are the most commonly prescribed drugs for OA patients for pain control^3,36^. Finally, knee OA is a major cause of disability and muscle weakness in the elderly and causes physical activity restrictions. Reduced physical activity is an important risk factor for CVD^18–20^.

According to the results of this study, subjects with knee OA had a 20% and 29% higher risk of MI and stroke than comparisons, respectively. In the baseline analysis, the knee OA cohort had a higher rate of comorbidities such as hypertension, diabetes, and dyslipidemia compared to the comparison cohort. However, after adjusting for 11 confounding factors including comorbidities, the still increased risk of CVD suggests that knee OA is independently associated with the occurrence of CVD. In addition, subgroup analysis confirmed that knee OA subjects under the age of 65 years, without diabetes or dyslipidemia had a higher risk of CVD. These results further support the strong association between knee OA and CVD.

Consistent with our results, in three population-based cohort studies, hip or knee OA increased the risk of developing CVD, and it was observed that the risk was further increased with the degree of disability caused by OA^2,37,38^. Also, in two systemic reviews and meta-analysis, Andrew et al. reported increased heart failure [relative risk (RR): 2.80; 95% CI 2.25–3.49] and ischemic heart disease (RR: 1.78; 95% CI 1.18–2.69)^21^, and Haoran et al. confirmed the CVD risk of OA patients increased by 24% compared to the comparison group^9^. The study of Rahman et al.^14^ and Ong et al.^39^ did not confirm the association between general OA or hip/knee OA and stroke, but our study did observe the relation between knee OA and stroke.

It may vary depending on whether it is caused by CVD or by all causes, there is a controversy over the mortality. Some studies have provided an increase in mortality due to CVD in patients with OA^38,40^. In contrast, there also exists a study in which hip/knee OA does not increase the risk of mortality^41,42^, and in our study, there was no evidence of an increase in all-cause death in knee OA patients. However, although not statistically significant, HR estimates of all-cause death were also higher in the knee OA cohort than in the general population.

Healthy physical activity is known as the keystone of CVD prevention and management. Exercise has been widely documented to modify potential risk factors in CVD such as obesity, diabetes, and hypertension by improving weight, glucose, and lipid control and lowering blood pressure at rest^43–45^. Similarly, in this study, it was observed that the risk of CVDs increased in patients with knee OA who had poor exercise habits. However, despite the widely known general benefits of exercise, there have been concerns and debates over whether exercise may cause pain or worsen symptoms in OA patients. In particular, this concern is more pronounced in knee OA because it is a major cause of elderly disability. But growing evidence dispels this concern. Regular physical activity and exercise are not the cause of OA unless injured, but rather have the benefit of reducing pain and disability^46–48^.

Our results warn against the ominous scenario of CVD that may come in non-exercise knee OA patients while demonstrating the CVD prevention effectiveness of exercise in knee OA patients. It is also noteworthy that the prevention effect has been confirmed even with 20–30 min of exercise once a week.

Aging is a strong independent predictor shared by OA and cardiovascular events. Therefore, the subgroup analysis of our study confirmed that under the age of 65 years with knee OA had a low CVD incidence, but paradoxically increased the CVD risk significantly. If OA develops at a young age, the duration of the disease increases, and if it is accompanied by poor exercise behavior, it seems that the risk of critical complications such as CVD will increases. These results advocate that lifestyle changes including steady exercise and modification of risk factors should be recommended for young knee OA patients.

This study is a large population-based cohort study evaluating the association of knee OA on CVD and mortality, and the first study to determine the effect of differences in exercise behavior in patients with knee OA on CVD. Nevertheless, there are some limitations to our study. First, due to the study design of the retrospective observational cohort study, the association between knee OA and CVD can be confirmed, but the causal relationship cannot be revealed. Second, the level of exercise collected by the self-report questionnaire cannot ignore the effect of recall bias. Also, since the contents of the questionnaire consisted mainly of aerobic exercise, information on the non-aerobic exercise performed by the subject was limited. Third, in the sensitivity analysis of participants with MI only, the difference identified in the crude analysis between knee OA and the comparison cohorts was not confirmed after adjusting for 11 confounding variables. Therefore, it is not possible to exclude the possibility that various variables influenced the study results, so careful interpretation of the results is required. Fourth, because our study was based on claims data, we could not confirm the information on the grade of knee OA. Fifth, the use of drugs that could affect the development of CVD, such as NSAIDs, was not considered. Sixth, since our research was conducted with the data of the Korean National Health Insurance Service, there may be limitations in generalizing our research results to other ethnic groups.

Knee OA is a prevalent disease in the elderly population, and both CVD and knee OA are medical burdensome diseases. Encouraging knee OA patients to maintain healthy exercise behavior helps to reduce the risk of CVD as well as knee joint health. Further research is needed in future studies on the optimal exercise method and intensity to improve CVD and mortality in knee OA patients. In addition, its effect on health costs is needed to be investigated in the future.

## Conclusion

Our findings highlight that knee OA may be associated with an increased risk of CVD in a nationally representative population-based context. It also suggests that lack of exercise in knee OA patients may have a detrimental effect on CVD development. But, there was no association between knee OA and all-cause death. Clinicians should recommend exercise from the perspective of CVD prevention in knee OA patients, and this intervention may be warranted at a younger age.

## Supplementary Information

Supplementary Information 1.

Supplementary Information 2.

## Data Availability

The data presented in this study are available in the main article.
