# Supplementary material for: Association between knee osteoarthritis and the risk of cardiovascular disease and the synergistic adverse effects of lack of exercise
Source: Sci Rep. 2023 Feb 16;13:2777. doi: 10.1038/s41598-023-29581-1 (PMC9935498; doi:10.1038/s41598-023-29581-1)
Supplement: Supplementary file 2 — Supplementary Information 2. [file 41598_2023_29581_MOESM2_ESM.docx]

| **S1 Table. Definition of comorbidities** | | |
| --- | --- | --- |
| **Comorbidities** |  | **ICD-10-CM code and definition** |
| Hypertension ^a^ | | I10-I13, I15 and minimum one prescription of anti-hypertensive drug (thiazide, loop diuretics, aldosterone antagonist, alpha-/beta-blocker, calcium-channel blocker, angiotensin-converting enzyme inhibitor, and angiotensin II receptor blocker) ; or systolic BP (SBP) ≥ 140 mmHg or diastolic BP (DBP) ≥ 90 mmHg |
|  |  |  |
| Type 2 diabetes mellitus ^a^ | | E11-E14 and minimum one prescription of anti-diabetic drugs (sulfonylureas, metformin, meglitinides, thiazolidinediones, dipeptidyl peptidase-4 inhibitors, α-glucosidase inhibitors, and insulin) ; or fasting plasma glucose value ≥126 mg/d |
|  |  |  |
| Dyslipidemia ^a^ | | E78 and use of lipid-lowering agents ; or fasting total cholesterol ≥ 240 mg/dL |
|  |  |  |
|  |  |  |
| Chronic kidney disease |  | estimated GFR < 60 mL/min/1.73 m^2^ |
|  |  |  |

ICD-10-CM=the International Classification of Disease

^a^ Defined by combination of diagnosis identified through ICD-10 code (≥ 1 diagnosis during hospitalization or at the outpatient clinic, in the previous one year) and claim data of related drugs or measured value of health examination

**S2 Table. Sensitivity analysis for risk of myocardial infarction (except stroke) and stroke (except MI) in patients with knee OA and control cohorts**

|  |  | **N** | **Event** | **Duration,  Person-years** | **IR  (per 1,000)** | **HR (95% CI)** | | |
| --- | --- | --- | --- | --- | --- | --- | --- | --- |
|  |  |  |  |  |  | **Model 1** | **Model 2** | **Model 3** |
| **MI (except stroke)** | |  |  |  |  |  |  |  |
|  | Controls | 193,894 | 2,162 | 1,366,460 | 1.58 | 1 (ref.) | 1 (ref.) | 1 (ref.) |
|  | Knee OA | 7572 | 107 | 55347.03 | 1.93 | 1.22 (1.01, 1.49) | 1.18 (0.97, 1.44) | 1.16 (0.96, 1.42) |
| **Stroke (except MI)** | |  |  |  |  |  |  |  |
|  | Controls | 193,894 | 5,942 | 1,366,460 | 4.35 | 1 (ref.) | 1 (ref.) | 1 (ref.) |
|  | Knee OA | 7,572 | 373 | 55,347 | 6.74 | 1.56 (1.41, 1.74) | 1.28 (1.15, 1.43) | 1.27 (1.14, 1.41) |

Model 1: no adjustment; Model 2: adjusted for age and sex; Model 3 : adjusted for age, sex, income, hypertension, dyslipidemia, smoking, drinking, exercise, BMI, glucose, and GFR

OA, osteoarthritis; CVD, cardiovascular disease; MI, myocardial infarction; IR, incidence rate; HR, hazard ratio; CI, confidence interval
